# Bioinformatic exploration of OLFML2B overexpression in gastric cancer base on multiple analyzing tools

**DOI:** 10.1186/s12885-019-5406-x

**Published:** 2019-03-13

**Authors:** Jiaxin Liu, Zhao Liu, Xiaozhi Zhang, Tuotuo Gong, Demao Yao

**Affiliations:** 1 Department of Geriatric Surgery, First Affiliated Hospital of Xi’an Jiaotong University, Xi’an, Shanxi 710061 People’s Republic of China; 2 Department of Oncology Surgery, First Affiliated Hospital of Xi’an Jiaotong University, Xi’an, Shanxi 710061 People’s Republic of China; 3 Department of Radiotherapy, First Affiliated Hospital of Xi’an Jiaotong University, Xi’an, Shanxi 710061 People’s Republic of China

**Keywords:** OLFML2B, Gastric cancer, Bioinformatic, Diagnosis, Prognosis

## Abstract

**Background:**

Gastric cancer (GC) is one of the most commonly occuring gastrointestinal tumor types, and early diagnosis and operation have a notable effect on the prognosis of patients. Although certain markers, including HER2, VEGER-2, ERCC1 and Bcl-2, have been utilized in clinical practise to screen out new patients with GC, the results of using these markers remains poor. The role of olfactomedin-like 2B (OLFML2B) in GC, as a member of the olfactomedin domain-containing proteins family, is remain unclear.

**Methods:**

In the present study, we assessed the expression of OLFML2B, including mRNA and protein level, by using The Cancer Genome Atlas (TCGA) database and 13 pairs of clinical samples between GC and NG tissues. The correlation between expression of OLFML2B and prognosis of GC was evaculated by the Kaplan-Meier plotter and OncoLnc online tools. In addition, mechanism analysis of OLFML2B in GC was explored thought bioinformatic tools, including cBioPortal and FunRich software.

**Results:**

In our study, the mRNA expression of OLFML2B in GC both TCGA database and clinical samples was consistently revealed to be significantly higher compared with that in NG tissues (*P* < 0.0001 for TCGA database and *P* = 0.0034 for clinical samples), and high OLFML2B expression was found in 9 (69.23%) of 13 clinical GC by immunohistochemistry and was positively correlated with the depth of tumor invasion and clinical stage (TNM). In addition, the AUC for a ROC of 0.867 indicated a moderate diagnostic ability of OLFML2B for GC. Survival analysis from the Kaplan-Meier plotter (*P* = 2.6 × 10^− 6^) and OncoLnc (*P* = 0.00276) revealed that the high expression of OLFML2B was associated with a short overall survival. Futhermore, 5% (24/478) alterations of OLFML2B were identified from cBioPortal, and among them, missense mutation (14/478) was the primary type. The results from FunRich revealed that OLFML2B participated in mediating multiple biological processes including cell growth and maintenance, regulation of the cell cycle, apoptosis and cell communication through multiple signaling pathways including the M/G1 transition pathway, post-translational protein modification and DNA replication pre-initiation.

**Conclusions:**

Taken together, it could be deduced that OLFML2B may act as an oncogene in the development of GC and the overexpression of OLFML2B in GC may be used as a novel diagnostic and prognostic target for GC.

## Background

Gastric cancer (GC) is a malignant tumor type that seriously threatens human health, whose mortality rate ranks second globally [1]. Approximately 1,000,000 new cases [2] of GC occur every year. The existing treatments of GC include surgery, radiotherapy, chemotherapy and other adjuvant therapies, however, the prognosis of patients with GC remains poor [3, 4]. In comparison, the 5-year survival rate of patients with early GC following radical resection is up to 90% [5]. Therefore, the present study aimed to determine an effective diagnostic and therapeutic target to improve the survival time of patients with GC.

The human olfactomedin-like 2B (OLFML2B) gene is located on chromosome 1q23.3 [6], consists of at least 8 exons and spans 40.7 kb, and is mainly expressed in the adult retina [7]. The encoded protein belongs to the olfactomedin domain-containing proteins family. The members of this family share a common feature in that the C-terminus of the proteins contains a 250 amino acid region, which is named as the olfactomedin region [8–12]. In addition, OLFML2B protein contains an unique Ser/Thr-rich region which was absent in other family members [13, 14] and is able to bind to chondroitin sulphate-E or heparin in the extracellular matrix [14]. However, the functions of OLFML2B in GC remain unclear.

Until now, certain specific markers, including human epidermal growth factor receptor-2 [15, 16], vascular endothelial growth factor receptor 2 [17], excision repair cross-complementation group 1 [18], B-cell lymphoma-2 and Ki-67 [19] have been used for the diagnosis of patients with GC. However, the majority of them were not able to accurately reflect diagnostic value and therapeutic efficiency. Therefore, we evaluated the expression of OLFML2B both TCGA database and clinical samples to reveal its clinical significance in GC, whilst investigating the status of OLFML2B from the cBioPortal to further reveal the molecular mechanisms of OLFML2B in the pathogenesis of GC.

## Methods

### Microarray data analysis

Original data between GC and normal gastric (NG) tissues were downloaded from the Gene Expression Omnibus (GEO; https://www.ncbi.nlm.nih.gov/) [20] database and three gene expression profiles (GSE54129, GSE33651 and GSE13911) were selected. The array data of GSE54129 (GPL570) comprised of 111 GC and 21 NG tissue samples [21]. GSE33651 (GPL2895) included 40 GC and 12 NG tissue samples [22]. GSE13911 (GPL570) consisted of 38 GC and 31 NG tissue samples [23]. GEO2R (http://www.ncbi.nlm.nih.gov/geo/geo2r/), an online analysis tool [24] that is able to compare two cohorts of samples in identical conditions, was applied to screen differentially expressed genes (DEGs) between GC and NG tissues. The adjusted *P* values were applied to correct the occurrence of false positive results using the Benjamini and Hochberg false discovery rate method [25] by default. The fold change (FC) of OLFML2B was assessed by log transformation. |logFC| > 1 and adjusted *P* < 0.01 were defined as the screened threshold.

### OLFML2B gene filtering

Each dataset selected from the GEO database was analyzed using the GEO2R tool, and the overlapping genes between GSE54129, GSE33651 and GSE13911 were discovered by Venny 2.1.0 (http://bioinfogp.cnb.csic.es/tools/venny/index.html) [26] that is able to analyze the intersection of multiple samples online. The screened process was as follows: i) Genes associated with GC survival were preferentially selected using Kaplan-Meier plotter [27] or OncoLnc [28], and *P* < 0.01 was set as the cut-off criterion; and ii) There was a significant difference between the GC and NG tissues.

### TCGA database analysis

To figure out the expression of OLFML2B in GC, a total of 384 GC and 37 NG tissue samples that contained the expression of OLFML2B were collected from TCGA database (http://cancergenome.nih.gov/). Additional clinical variables, including age, gender, tumor stage, lymph metastasis, distant metastasis and clinical stage according to the AJCC TNM stage [29], were analyzed to assess the association between the expression of OLFML2B and these parameters. Finally, the expression of OLFML2B between GC and NG tissues was analyzed using GraphPad Prism 5.01 software (GraphPad Software, Inc., La Jolla, CA, USA).

### Expression of OLFML2B in clinical samples

To further verify the mRNA expression of OLFML2B between GC and NG tissues, we collected 13 pairs of clinical cases from the First Affiliated Hospital of Xi’an Jiaotong University from February 2018 to April 2018. All patients (9 males and 4 females, the median age of 63, range 54–78 years) were diagnosed with GC by pathological examination and the RNA of each sample, including GC and NG tissues, was extracted according to TRIzol® protocol, The expression of OLFML2B was then evaluated by QuantiTect SYBR Green polymerase chain reaction (PCR) kits (Qiagen GmbH, Hilden, Germany) using Bio-Rad CFX Manager detection system, β-actin was used as an internal standard, The PCR cycle protocol used the following parameters: 95 °C for 30 s, 40 cycles of 5 s at 95 °C and 30 s at 58 °C. The fold-expression was calculated using the 2^-△△Ct^ method [30]. The primers used are as follow: OLFML2B: forward: 5′-AAC AGA CTC GCT GGG GAA AG-3′, reverse: 5′- CCC CGT GAT TGT GGA GAG AG T-3′; β-actin: forward: 5′-CCT TGC ACA TGC CGG AG-3′, reverse: 5′-GCA CAG AGC CTC GCC TT-3′. Ethical approval was provided by the First Affiliated Hospital of Xi’an Jiaotong University Ethics committee.

### Immunohistochemical staining

OLFML2B expression in protein level was detected by immunohistochemical (IHC). A total of 13 pairs of surgical specimens were paraffin-embedded and all tissue blocks were cut in a 4 μm-thick sections. The IHC was performed base on a published protocol [31]. The rabbit anti-OLFML2B antibody was provided by BIOSS (Beijing, China. 1:400 dilution).

The expression of OLFML2B in GC and NG tissues were evaluated according to the combined scores of the staining intensity and positive staining ratio of OLFML2B. The immunostaining was recorded based on the following standards: 1) staining intensity were transformed in different scores (no staining = 0, weak = 1, moderate = 2, strong = 3), and positive staining ratio of OLFML2B was designated from the range 0 (< 5%), 1 (5–50%), to 2 (> 50%). The final score of OLFML2B expression was divided into low expression (0–2) and high expression (3-4).

### Diagnostic and prognostic significance

To evaluate the effect of OLFML2B on the prognosis of GC, TCGA data was divided into different subgroups according to age (≥60 years/< 60 years), gender (male/female), tumor stage [29] (T2–4/T1), lymph node metastasis (N1–3/N0), distant metastasis (M1/M0), clinical stage [29] (II-IV/I) and expression of OLFML2B (mean value). Univariate and multivariate analysis based on a Cox proportional hazard regression model were used to detect the correlation between these elements and the prognosis of patients with GC. In addition, the Kaplan-Meier plotter (http://kmplot.com/) and OncoLnc (http://www.oncolnc.org/), online analysis tools, were also used to evaluate the effect of OLFML2B expression on the prognosis of gastric cancer. Furthermore, the diagnostic ability of OLFML2B in GC was detected using a reciever operating characteristic (ROC) curve by GraphPad Prism 5.01 software.

### Bioinformatic analysis

The status of OLFML2B in GC was detected by the cBioPortal OncoPrint (http://www.cBioPortal.org/index.do) [32, 33] and the impact of OLFML2B alteration on the prognosis of GC patients, including the overall survival (OS) and disease-free survival (DFS) was also examined. In addition, the co-expressed genes associated with OLFML2B were screened from Coexpedia (http://www.coexpedia.org/) [34] and potential biological processes and biological pathways were predicted by FunRich 2.1.2 software to explore the molecular mechanisms of OLFML2B in GC.

### Statistical analysis

All data were analysed using SPSS 17.0 (SPSS, Inc., Chicago, IL, USA). The association between the expression of OLFML2B and clinicopathological characteristics were evaluated using χ^2^ tests and Spearman’s correlation analysis. Univariate and multivariate analysis were based on Cox proportional hazard regression models. The variables showing significance (*P* < 0.05) by univariate analysis were adopted when multivariate Cox proportional hazards analysis was performed. The effect of OLFML2B on the prognosis of GC was evaluated by Kaplan-Meier plotter and OncoLnc. The expression of OLFML2B between GC and NG tissues was analyzed using a paired and unpaired Student’s t-test by Graphpad Prism 5.01 software. The diagnostic value of OLFML2B for GC was detected using a ROC curve. An area under curve (AUC) value of 0.5–0.7, 0.7–0.9 or 0.9–1.0 represented a low, moderate or high diagnostic ability, respectively. Experimental data were presented as the mean ± SD. *P* < 0.05 was considered to indicate a statistically significant difference.

## Results

### DEGs filtering

**Fig. 1:**
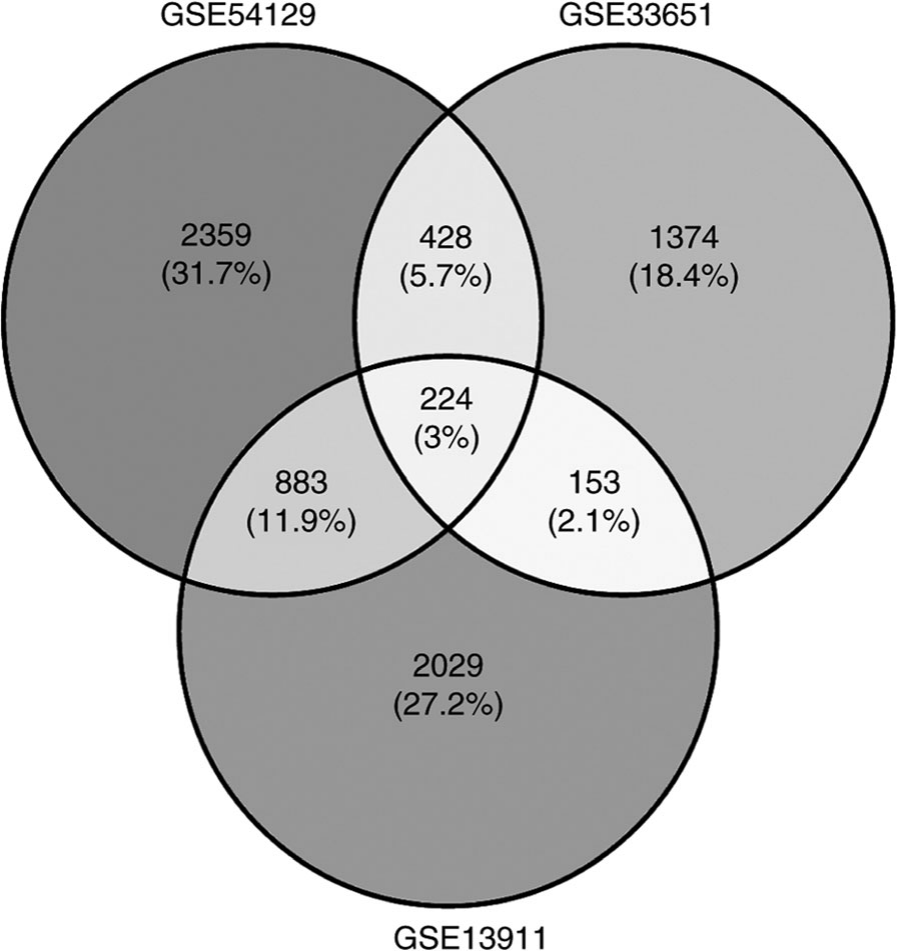
Venn diagram of DEGs between three datasets (GSE54129, GSE33651 and GSE13911) from GEO database by venny 2.1.0

Three GSE datasets were selected from GEO. By analysis conducted by the GEO2R online tool, the results revealed that GSE54129 consisted of 3894 DEGs, GSE33651 included 2179 DEGs and GSE13911 contained 3289 DEGs. There were 224 (3%) three-crossing genes identified by entering the DEGs into Venny 2.1.0 (Fig. 1). Of these genes, combining the screening criterias in Materials and Methods section with the results of corresponding articles [35–37] identified in Pubmed, OLFML2B was finally selected in the present study.

### OLFML2B expression

**Fig. 2:**
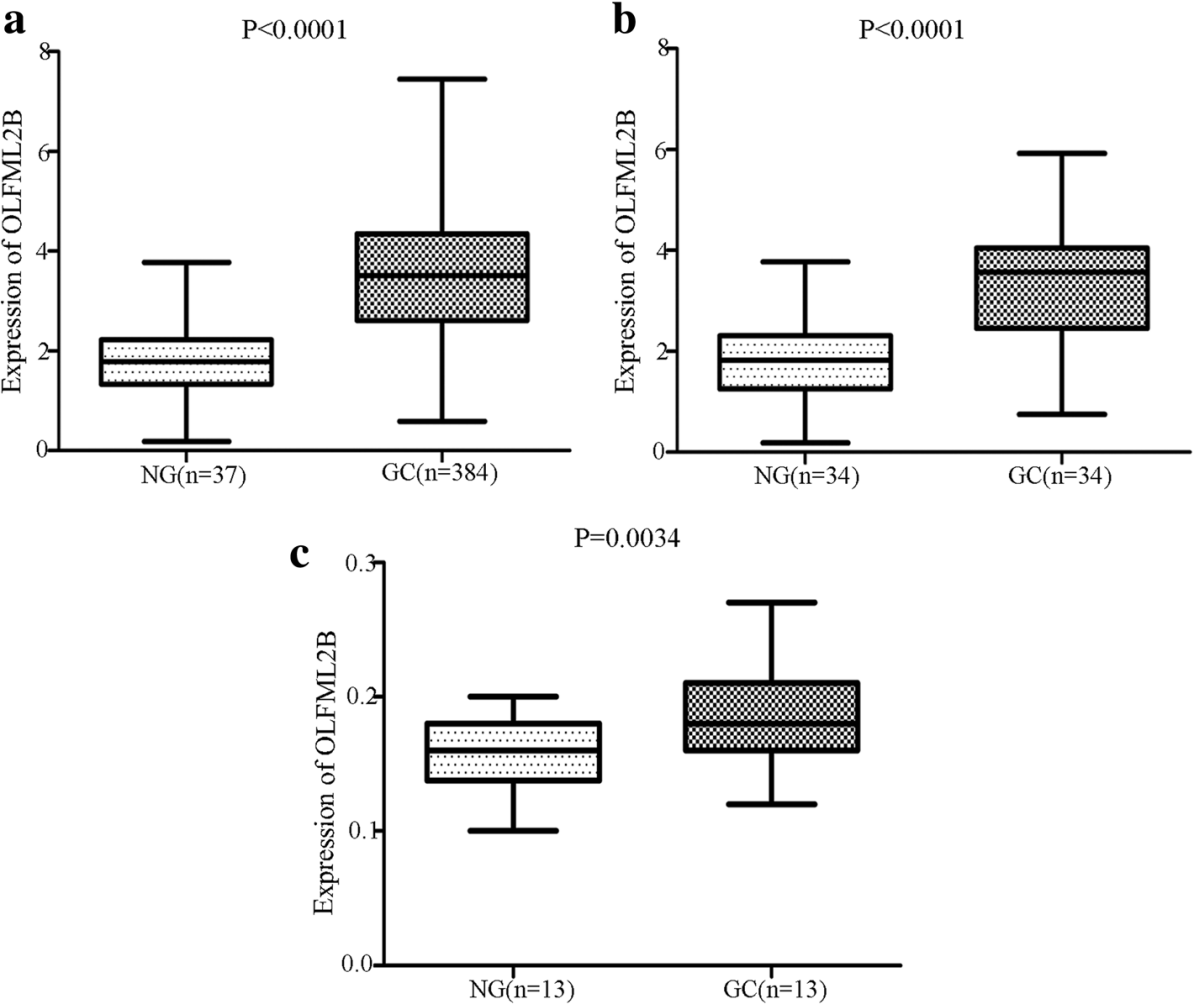
The expression of OLFML2B in GC. (**a**) The expression of OLFML2B was upregulated in 384 GC tissues compared with 37 NG tissues. (**b**) There was a similar trend on 34 pairs of GC tissues and the corresponding NG tissues. (**c**) A total of 13 pairs of clinical samples including GC tissues and NG tissues showed the same finding

**Fig. 3:**
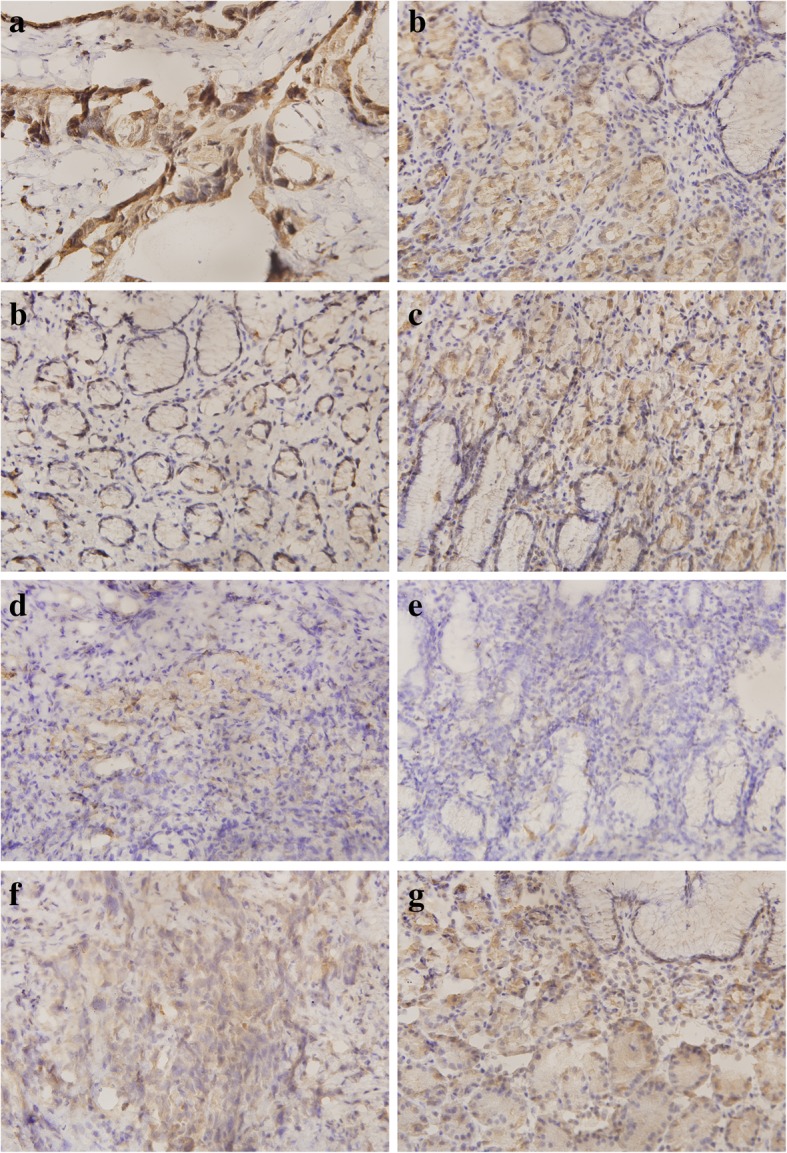
Immunohistochemical staining of OLFML2B in gastric specimens. Matched (**a**) and (**b**), high expression of OLFML2B in GC and low expression in NG tissues; Matched (**c**) and (**d**), low expression of OLFML2B in GC tissues and high expression in NG; Matched (**e**) and (**f**), low expression of OLFML2B in GC and NG; Matched (**g**) and (**h**), there was no statistical difference between GC and NG tissues. Left: GC tissues (× 400); Right: NG tissues (× 400)

χ^2^ tests analysis and Spearman’s correlation analysis revealed that OLFML2B expression was associated with tumor stage (*P* = 0.001, r = 0.175; Table1) and clinical stage (*P* = 0.027, r = 0.115; Table1), which were based on postoperative diagnosis. In addition, OLFML2B expression was significantly upregulated in 384 GC tissues compared with 37 NG tissues (*P* < 0.0001; Fig. 2a). Consistent results were obtained when the 34 matched pairs of GC and NG tissues were compared (Fig. 2b). Furthermore, a similar trend was also found in 13 pairs of clinical samples (Fig. 2c). As demonstrated in Fig. 3, OLFML2B expression in protein level was examined using IHC, the results showed that OLFML2B was overexpressed in 9 (69.23%) of 13 patients with gastric carcinoma and downregulated in 4 (30.77%) of 13 patients. Simultaneously, a positioning analysis revealed the OLFML2B was mainly focused on the cytoplasm of tumor cells.

### Survival and diagnostic analysis

**Fig. 4:**
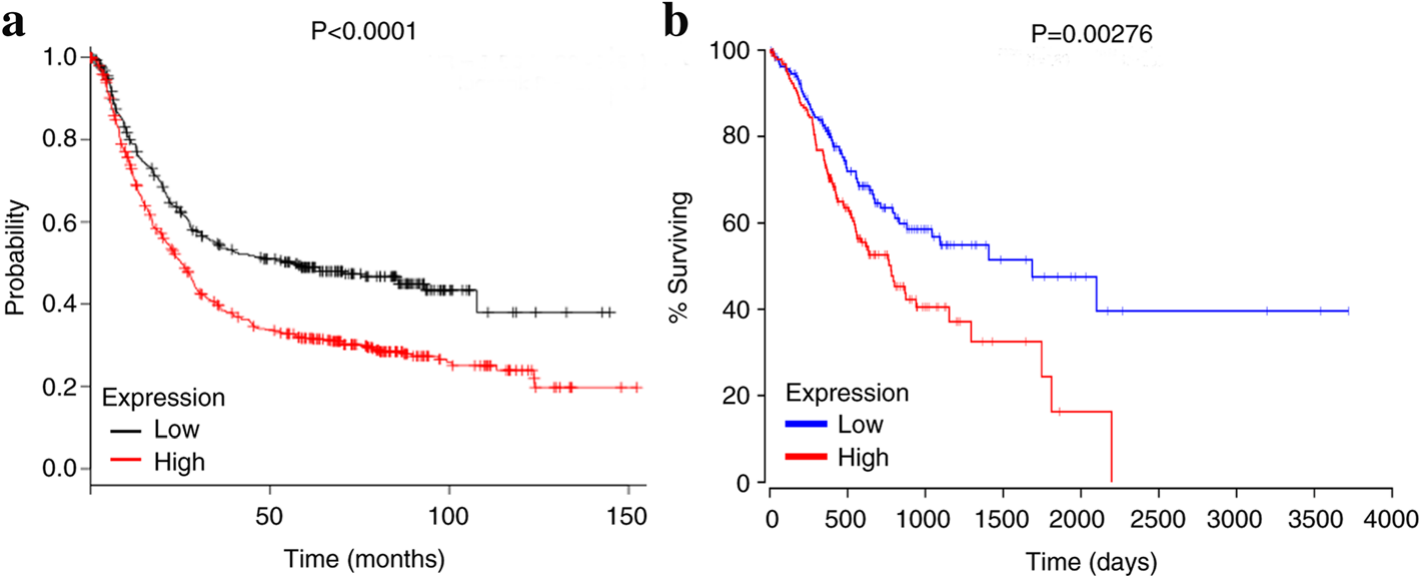
Survival analysis of OLFML2B in GC demonstrated that high OLFML2B expression was significantly associated with a reduced OS using Kaplan-Meier Plotter (**a**) and OncoLnc (**b**)

**Fig. 5:**
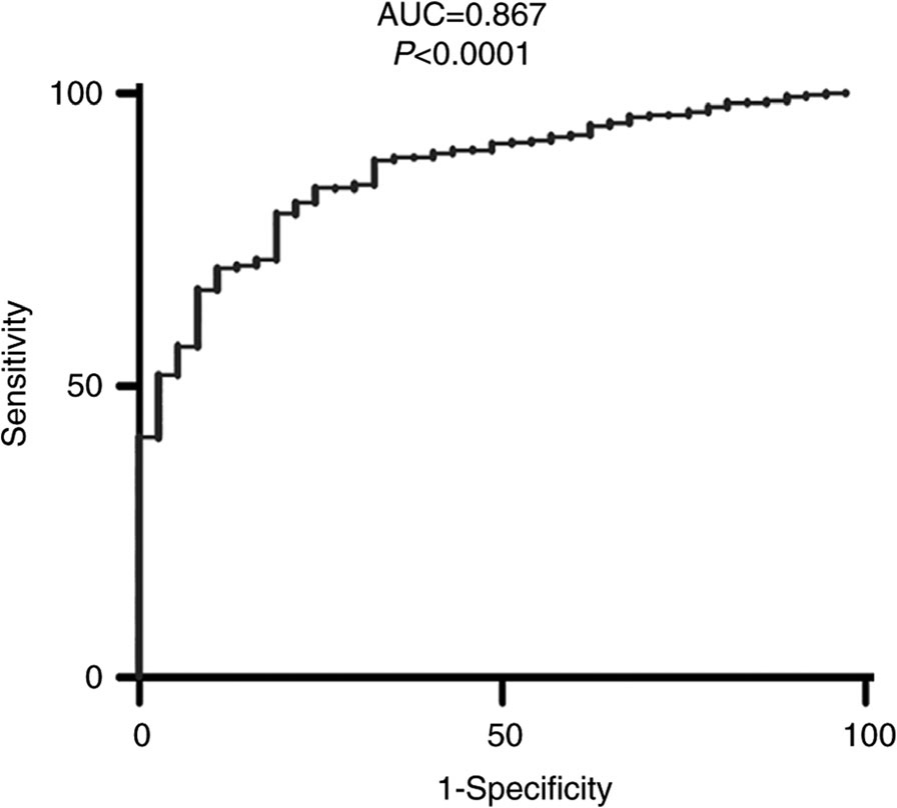
The ROC curve of OLFML2B expression in GC. The curve indicated that OLFML2B possessed a moderate diagnostic ability for GC (AUC =0.867, *P* < 0.0001)

Univariate Cox regression analysis confirmed that the OS of GC was significantly associated with age, lymph node metastasis and distant metastasis (Table2). However, multivariate analysis did not reveal an association between the expression of OLFML2B and the prognosis of GC (Table 3). In addition, the OS of patients with GC was analysed using Kaplan-Meier plotter and OncoLnc, and the results revealed that high OLFML2B expression was significantly associated with a shorter OS for all patients with GC [hazard ratio = 1.56, 95% CI (1.29–1.88); *P* = 2.6 × 10^− 6^; Fig. 4a]. The results by OncoLnc also revealed the same trend (*P* = 0.00276; Fig. 4b). Finally, a ROC curve (X-axis: 1-Specificity; Y-axis: Sensitivity) was used to estimate the diagnostic value of OLFML2B in GC. An AUC value of 0.867 revealed a moderate diagnostic value (*P* < 0.0001; Fig. 5).

### Bioinformatic analysis

**Fig. 6:**
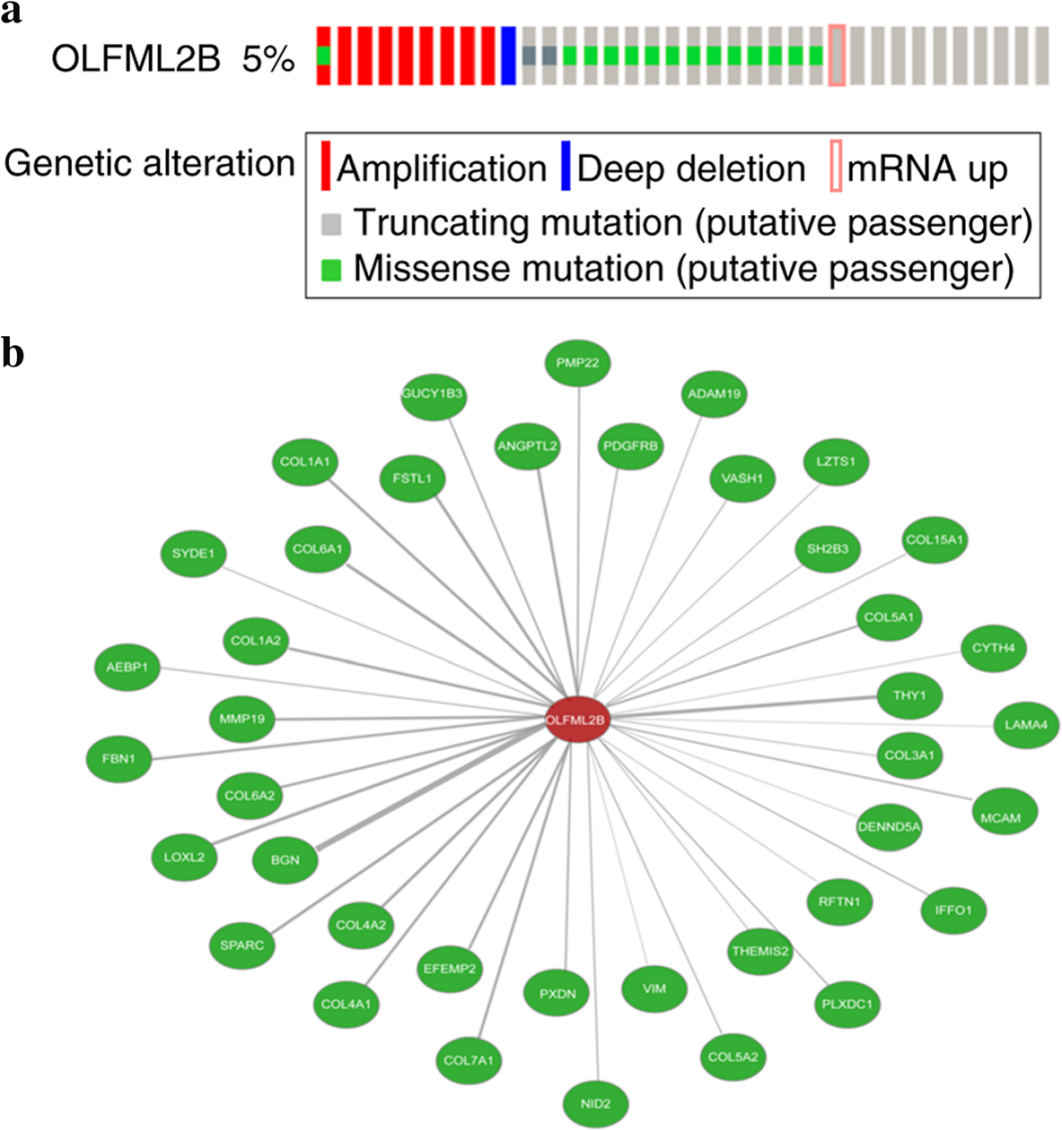
Alteration of OLFML2B and neighboring genes in GC. (**a**) A total of 5% (24/478) of GC cases exhibited OLFML2B alteration. (**b**) Network of OLFML2B and neighboring genes was anaysed by Coexpedia

**Fig. 7:**
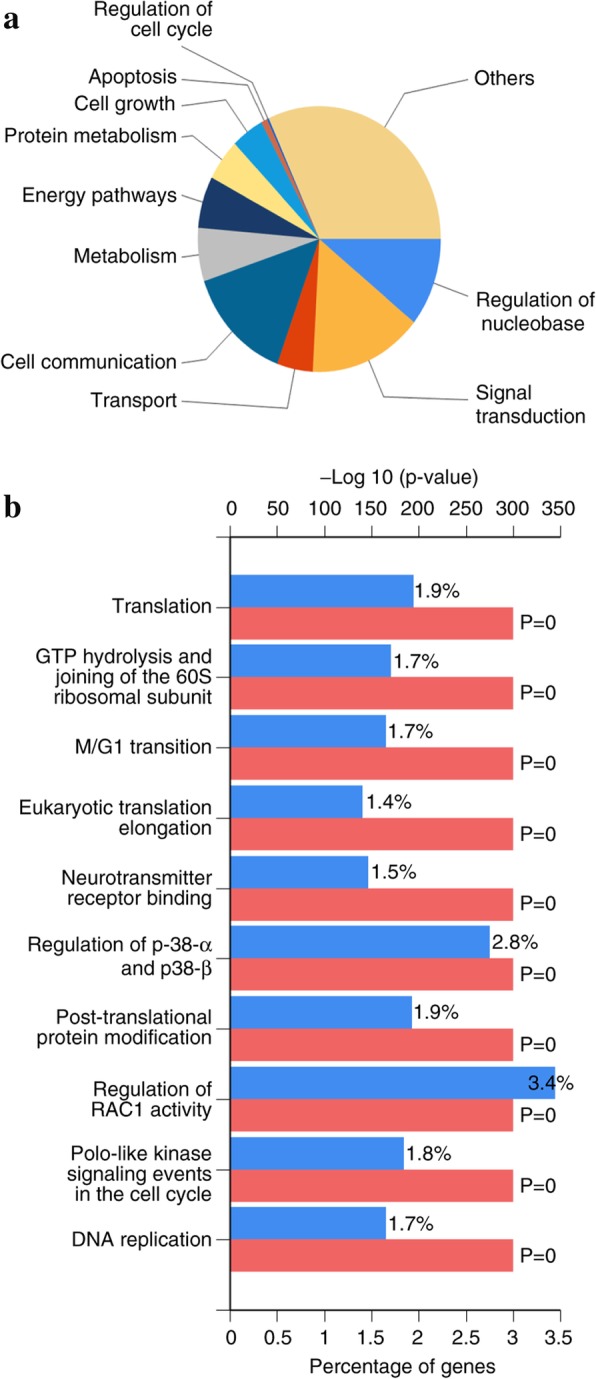
Potential biological processes (**a**) and biological pathways (**b**) involved in GC were identified by FunRich

**Fig. 8:**
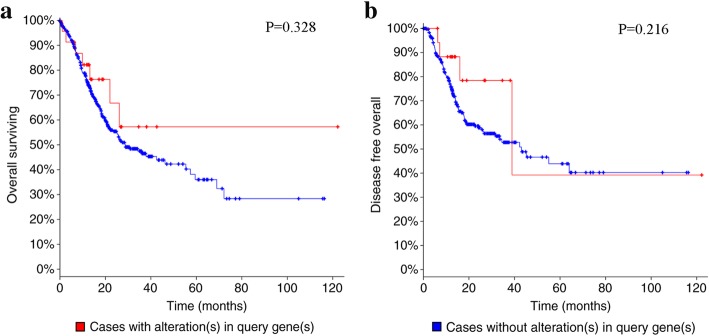
Analysis of the overall survival (**a**) and disease-free survival (**b**) of GC patients with and without OLFML2B alteration. No statistically significant difference was identified

The alteration of OLFML2B in 478 cases of GC available in cBioPortal were investigated. The result revealed that 5% (24/478) of cases of GC exhibited OLFML2B alteration, including missense mutation (14/478), amplification (9/478), mRNA upregulation (1/478) and deep deletion (1/478) (Fig. 6a), in addition, a number of neighboring genes in stomach neoplasms that were associated with OLFML2B were invested from Coexpedia to understand the molecular mechanism of GC (Fig. 6b). The network reflected interactions between OLFML2B and a total of 40 other genes, mainly including biglycan, Thy-1 cell surface antigen and matrix metallopeptidase 19 (MMP19). Furthermore, the biological processes and biological pathways of OLFML2B in GC were investigated. The result demonstrated that OLFML2B participated in mediating multiple biological processes including cell growth and maintenance, regulation of cell cycle, apoptosis and cell communication through multiple signaling pathways including the M/G1 transition pathway, post-translational protein modification and DNA replication pre-initiation (Fig. 7). Simultaneously, OS and DFS analysis was performed to compare the prognosis of patients with GC, with and without OLFML2B alteration. However, the result did not reveal a statistically significant difference (*P* = 0.328 for OS; *P* = 0.216 for DFS; Fig. 8).

## Discussion

GC is a type of gastrointestinal tumor that may occur in any section of the stomach [38]. It has been confirmed that GC originates from the gastric mucosa epithelium, and pathological results confirm that the majority of GCs are a type of adenocarcinoma [38]. Although gastroscopy has been used to detect early GC, the results were poor, therefore it is necessary to screen out novel diagnostic and prognostic targets for GC.

The olfactomedin domain-containing proteins family included 13 members in mammals, the functions of them remain unclear. The OLFML2B protein was originally identified in ganglion cells, inner nuclear layers, the inner segment of the photoreceptor layer and the retinal pigmented epithelium. It exerts an important function by specifically binding to chondroitin sulphate-E or heparin in the extracellular matrix [14]. To date, no studies have elaborated on the diagnostic and prognostic value of OLFML2B for GC. The present research, to the best of our knowledge, is the first one to systematically survey the clinical significance of OLFML2B in GC.

As showed in Table 1, χ^2^ tests was performed to assess the correlation between OLFML2B expression (low & high) and clinicopathological characteristics. The result demonstrated that high expression of OLFML2B predicted deeper tumor invasion and later clinical stage, but it was not correlated with lymph node metastasis and distant metastasis. The main reasons were considered as follow: 1) There were multiple factors involved in the occurrence and progress of GC, including oncogene activation, inactivation of tumor suppressor genes, H.pylori infection, and so on. When χ^2^ tests was performed, it couldn’t exclude influence of other factors. 2) 376 samples from TCGA database were included in our study, it couldn’t too enough to explain the correlation between OLFML2B expression and clinicopathological characteristics, so more samples need to be collected to evaluate their correlation in the future.

**Table 1:** Clinical association between OLFML2B expression and clinicopathological variables in GC patients

Variable	Number	OLFML2B expression		χ^2^-test	Correlation	
		Low	High	*P*-value	*r*	*P*-value
Age	371^a^				0.008	0.872
≥ 60	255	123	132	0.872		
< 60	116	57	59			
Sex					0.044	0.392
Male	243	113	130	0.391		
Female	133	68	65			
Tumor stage					0.175	0.001
T2–4	356	164	192	0.001		
T1	20	17	3			
Lymph metastasis					0.025	0.626
Yes	258	122	136	0.625		
No	118	59	59			
Distant metastasis					0.016	0.764
Yes	35	16	19	0.763		
No	341	165	176			
Clinical stage	372^a^				0.115	0.027
II-IV	321	148	173	0.027	0	
I	51	32	19			

^a^Complete data was unavailable in TCGA database

In addition, the analysis from TCGA database indicated that OLFML2B had a significantly higher expression in GC compared with in NG tissues, and the same trend was observed in 13 pairs of clinical samples by qRT-PCR and IHC test. Thus, OLFML2B might serve as an oncogene in the development of GC. It was inferred that OLFML2B may accelerate the growth of GC by promoting the proliferation of GC cells. Further investigations in vivo and in vitro are required in order to assess the molecular mechanisms of OLFML2B in GC.

As demonstrated in Tables 2 and 3, univariate and multivariate analysis did not select OLFML2B expression as independent prognostic factor. The causes were explained as follow: 1) There were many factors that influenced the prognosis of GC, the common ones were listed in current study and other mixed facters couldn’t be excluded. 2)Analysis from Kaplan-Meier plotter and OncoLnc revealed that high OLFML2B expression was significantly associated with a shorter OS for all patients with GC. Considering these facts it can be deduced from our result OLFML2B expression have the clinical utility for predicting prognosis in patients with GC.

**Table 2:** Univariate analysis of prognostic factors of GC

Variable	OS		
	Hazard ratio	95% CI	*P*-value
Age(<60/≥60)	1.557	(1.034,2.346)	0.034
Gender (Male/Female)	1.468	(0.984,2.189)	0.060
Tumor size (T2–4/T1)	3.503	(0.865,14.188)	0.079
Lymph metastasis (Yes/No)	1.689	(1.095,2.607)	0.018
Distant metastasis (Yes/No)	2.265	(1.337,3.889)	0.002
Clinical stage (II- IV/I)	1.558	(0.872,2.783)	0.134
OLFML2B expression (High/Low)	1.331	(0.929,1.907)	0.119

**Table 3:** Multivariate analysis of prognostic factors of GC

Variable	OS		
	Hazard ratio	95% CI	*P*-value
Age, years (≥60/< 60)	1.669	(1.095,2.542)	0.017
Gender (Male/Female)	1.313	(0.875,1.970)	0.189
Tumor size (T2–4/T1)	2.485	(0.547,11.298)	0.239
Lymph metastasis (Yes/No)	1.676	(0.947,2.966)	0.076
Distant metastasis (Yes/No)	2.293	(1.345,3.909)	0.002
Clinical stage (II-IV/I)	0.835	(0.376,1.854)	0.659
OLFML2B expression (High/Low)	1.198	(0.830,1.727)	0.334

To date, multiple methods were used to diagnose GC, among which serum marker was the more convenient than others, the common markers in clinical included serum CA199 and CEA. However, the specificity of them for diagnosing GC was related to the depth of tumor invasion and individual differences. Our results demonstrated OLFML2B had a significant diagnostic value for GC (AUC = 0.867; *P* < 0.0001). There is currently no reports on the association of OLFML2B expression and these markers. Therefore, combined detection of multiple markers may improve the diagnostic ratio of GC in future.

However, whether OLFML2B is effective for clinical application as replacements for or in addition to the prognostic parameters currently in use is still unclear, and further investigation is called for to identify whether combined detection of OLFML2B together with some of these other molecules would be valuable in improving prognostic effectiveness.

The alterations to OLFML2B in GC were detected to identify potential mechanisms in GC. The result indicated that 5% of OLFML2B contained alterations, among which the missense mutation was the primary type of alteration, followed by amplification, mRNA upregulation and deep deletion. It was hypothesised that the missense mutation of OLFML2B was associated with the unique Ser/Thr-rich region, and the missense mutation may facilitate the transcription of OLFML2B. Futhermore, OLFML2B protein may specifically bind to chondroitin sulphate-E in the extracellular matrix, which may be associated with the size of the tumor in clinical pathology. Then, the coexpressed genes in stomach neoplasms were examined by Coexpedia, and a total of 40 genes were identified. Thus, it could be deduced that the functions of OLFML2B in GC were achieved by interacting with other genes, particularly MMP19, which indicated that OLFML2B might be involved in the epithelial mesenchymal transition process. In addition, a number of potential biological processes associated with OLFML2B, particularly cell growth and apoptosis, were predicted by FunRich software, which could be infered that these processes were unbalanced in GC, and further studies were required to verify the present results. However, although the bioinformatics analysis provided us a novel direction for future research, further studies need to be done to validate the functions of OLFML2B in GC.

## Conclusions

Taken together, OLFML2B was significantly overexpressed in GC and showed a moderately diagnostic and prognostic value for GC. It is likely to become a newly diagnostic and prognostic target for GC in the future.

## Abbreviations

AUC: An area under curve
DEGs: Differentially expressed genes
DFS: Disease-free survival
FC: Fold change
GC: Gastric cancer
MMP19: Matrix metallopeptidase 19
NG: Normal Gastric
OLFML2B: Olfactomedin-like 2B
OS: Overall survival
ROC: receiver operating characteristic
TCGA: The Cancer Genome Atlas
