# Correction to: Bioinformatic exploration of OLFML2B overexpression in gastric cancer base on multiple analyzing tools

**DOI:** 10.1186/s12885-019-5937-1

**Published:** 2019-08-01

**Authors:** Jiaxin Liu, Zhao Liu, Xiaozhi Zhang, Tuotuo Gong, Demao Yao

**Affiliations:** 1 Department of Geriatric Surgery, First Affiliated Hospital of Xi’an Jiaotong University, Xi’an, Shanxi 710061 People’s Republic of China; 2 Department of Oncology Surgery, First Affiliated Hospital of Xi’an Jiaotong University, Xi’an, Shanxi 710061 People’s Republic of China; 3 Department of Radiotherapy, First Affiliated Hospital of Xi’an Jiaotong University, Xi’an, Shanxi 710061 People’s Republic of China

**Correction to: BMC Cancer (2019) 19:227**

**https://doi.org/10.1186/s12885-019-5406-x**

Following publication of the original article [1], the authors reported that Fig. 3 was incorrectly published with two “b” labels. The corrected figure is given below.

**Fig. 3:**
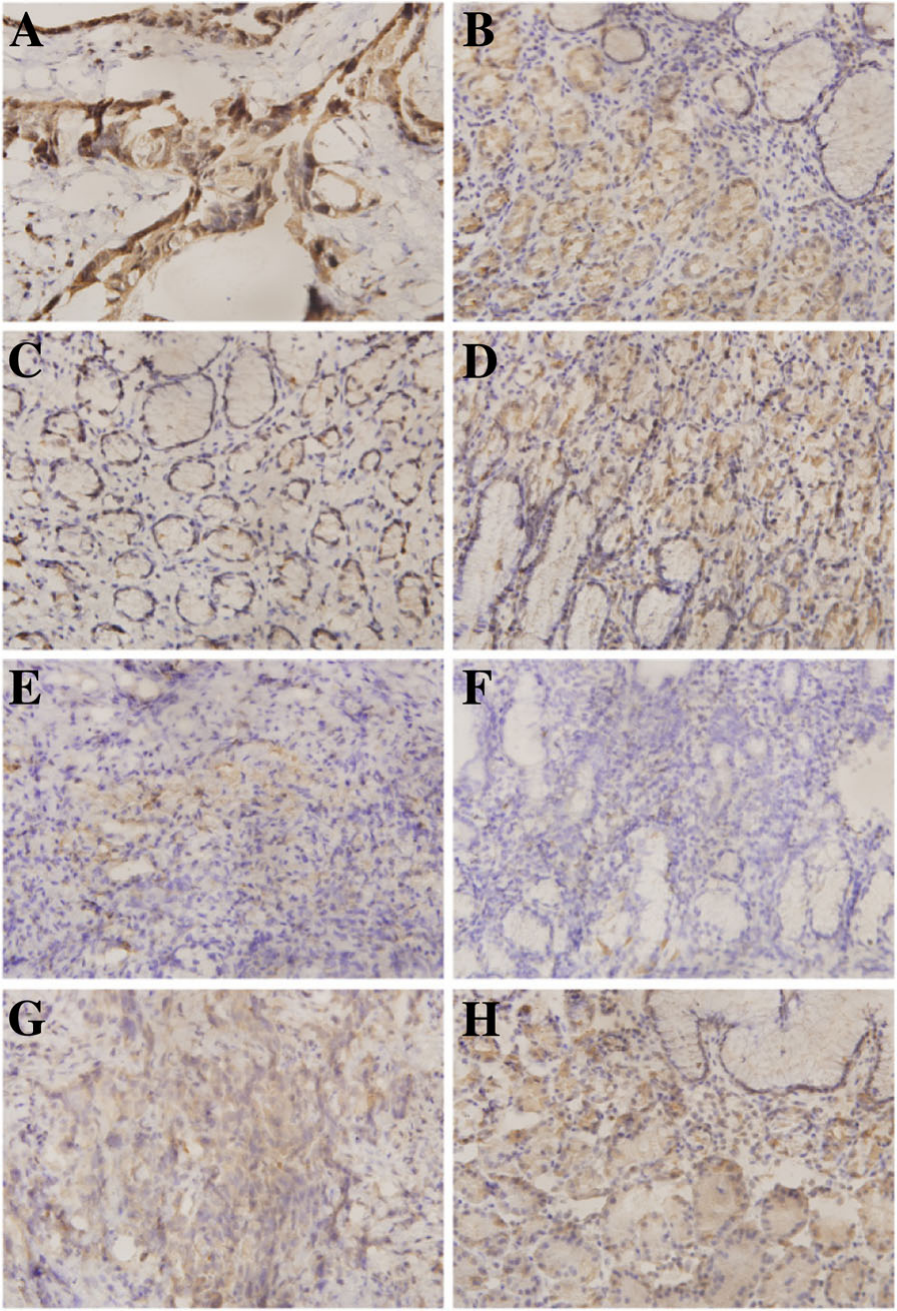
Immunohistochemical staining of OLFML2B in gastric specimens. Matched (**a**) and (**b**), high expression of OLFML2B in GC and low expression in NG tissues; Matched (**c**) and (**d**), low expression of OLFML2B in GC tissues and high expression in NG; Matched (**e**) and (**f**), low expression of OLFML2B in GC and NG; Matched (**g**) and (**h**), there was no statistical difference between GC and NG tissues. Left: GC tissues (× 400); Right: NG tissues (× 400)

The original article [1] has been corrected.
